# Virtually Transformative Experiences in Geriatric Education: Lessons Pre and Post Pandemic

**DOI:** 10.1093/geroni/igab046.234

**Published:** 2021-12-17

**Authors:** Pamela Saunders

**Affiliations:** Georgetown University, WASHINGTON, District of Columbia, United States

## Abstract

Georgetown University medical students have the option of selecting a two-week rotation in Geriatrics during their third-year. Since Fall 2019, the curriculum has included three immersive virtual reality (VR) labs: hearing & vision loss, Alzheimer’s disease, and end-of-life conversations created by Embodied Labs. The curricular goals include increasing empathy and sensitivity of learners to the perspective of older adults, decreasing ageism & stereotyping, and increasing clinical knowledge. In each lab, students are immersed in a live film, first-person point of view of an older adult. They interact with the immersive environment via gaze, voice, and natural hand motions. Pre-pandemic, students viewed the labs in-person using a commercial VR headset. Since the pandemic, March 2020, students accessed the VR labs through the virtual modality of Zoom. This abstract summarizes data on knowledge and attitudes examining differences in knowledge and attitudes pre and post-pandemic.

